# The Pathophysiology of Gestational Diabetes Mellitus

**DOI:** 10.3390/ijms19113342

**Published:** 2018-10-26

**Authors:** Jasmine F Plows, Joanna L Stanley, Philip N Baker, Clare M Reynolds, Mark H Vickers

**Affiliations:** 1 Department of Preventive Medicine, University of Southern California, Los Angeles, CA 90033, USA; plows@usc.edu; 2 Liggins Institute, University of Auckland, Auckland 1023, New Zealand; jostnly@gmail.com (J.L.S.); c.reynolds@auckland.ac.nz (C.M.R.); 3 University of Leicester, University Road, Leicester LE1 7RH, UK; philip.baker@leicester.ac.uk

**Keywords:** gestational diabetes, pregnancy, pathophysiology, physiology, pathology, molecular

## Abstract

Gestational diabetes mellitus (GDM) is a serious pregnancy complication, in which women without previously diagnosed diabetes develop chronic hyperglycemia during gestation. In most cases, this hyperglycemia is the result of impaired glucose tolerance due to pancreatic β-cell dysfunction on a background of chronic insulin resistance. Risk factors for GDM include overweight and obesity, advanced maternal age, and a family history or any form of diabetes. Consequences of GDM include increased risk of maternal cardiovascular disease and type 2 diabetes and macrosomia and birth complications in the infant. There is also a longer-term risk of obesity, type 2 diabetes, and cardiovascular disease in the child. GDM affects approximately 16.5% of pregnancies worldwide, and this number is set to increase with the escalating obesity epidemic. While several management strategies exist—including insulin and lifestyle interventions—there is not yet a cure or an efficacious prevention strategy. One reason for this is that the molecular mechanisms underlying GDM are poorly defined. This review discusses what is known about the pathophysiology of GDM, and where there are gaps in the literature that warrant further exploration.

## 1. Introduction

Gestational diabetes mellitus (GDM) is a common pregnancy complication, in which spontaneous hyperglycemia develops during pregnancy [1]. According to the most recent (2017) International Diabetes Federation (IDF) estimates, GDM affects approximately 14% of pregnancies worldwide, representing approximately 18 million births annually [2]. Risk factors include overweight/obesity, westernized diet and micronutrient deficiencies, advanced maternal age, and a family history of insulin resistance and/or diabetes. While GDM usually resolves following delivery, it can have long-lasting health consequences, including increased risk for type 2 diabetes (T2DM) and cardiovascular disease (CVD) in the mother, and future obesity, CVD, T2DM, and/or GDM in the child. This contributes to a vicious intergenerational cycle of obesity and diabetes that impacts the health of the population as a whole. Unfortunately, there is currently no widely-accepted treatment or prevention strategy for GDM, except lifestyle intervention (diet and exercise) and occasionally insulin therapy—which is only of limited effectiveness due to the insulin resistance that is often present. While emerging oral antidiabetics, such as glyburide and metformin, are promising, concerns remain about their long-term safety for the mother and the child [3,4]. Therefore, safe, effective, and easy-to-administer new treatments are sought. In order to develop such treatments, a thorough understanding of the pathophysiology of GDM is required. This review will discuss what is known about the pathophysiology of GDM and what has yet to be elucidated. In order to do so, a contextual summary of glucose regulation during normal pregnancy, classification of GDM, forms of GDM, risk factors for GDM, and consequences of GDM is first required.

### 1.1. Glucose Regulation during Healthy Pregnancy

During healthy pregnancy, the mother’s body undergoes a series of physiological changes in order to support the demands of the growing fetus. These include adaptations to the cardiovascular, renal, hematologic, respiratory, and metabolic systems. One important metabolic adaptation is in insulin sensitivity. Over the course of gestation, insulin sensitivity shifts depending on the requirements of pregnancy. During early gestation, insulin sensitivity increases, promoting the uptake of glucose into adipose stores in preparation for the energy demands of later pregnancy [5]. However, as pregnancy progresses, a surge of local and placental hormones, including estrogen, progesterone, leptin, cortisol, placental lactogen, and placental growth hormone together promote a state of insulin resistance [6]. As a result, blood glucose is slightly elevated, and this glucose is readily transported across the placenta to fuel the growth of the fetus. This mild state of insulin resistance also promotes endogenous glucose production and the breakdown of fat stores, resulting in a further increase in blood glucose and free fatty acid (FFA) concentrations [7]. Evidence in animals suggests that, in order to maintain glucose homeostasis, pregnant women compensate for these changes through hypertrophy and hyperplasia of pancreatic β-cells, as well as increased glucose-stimulated insulin secretion (GSIS) [8]. The importance of placental hormones in this process is exemplified by the fact that maternal insulin sensitivity returns to pre-pregnancy levels within a few days of delivery [9]. For reasons that will be explored in this review, the normal metabolic adaptations to pregnancy do not adequately occur in all pregnancies, resulting in GDM.

### 1.2. Classification and Prevalence of Gestational Diabetes

The American Diabetes Association (ADA) formally classifies GDM as “diabetes first diagnosed in the second or third trimester of pregnancy that is not clearly either preexisting type 1 or type 2 diabetes” [1]. However, the exact threshold for a diagnosis of GDM depends on the criteria used, and so far, there has been a lack of consensus amongst health professionals. It is now advised by the ADA, the World Health Organization (WHO), the International Federation of Gynaecology and Obstetrics, and the Endocrine Society, that the International Association of Diabetes and Pregnancy Study Group (IADPSG) criteria be used in the diagnosis of GDM [10]. The IADPSG criteria was developed based on the results of the Hyperglycemia and Adverse Pregnancy Outcomes (HAPO) Study—a large multinational and multicenter study of 23,000 pregnant women [11]. One major finding of the HAPO Study was a continuous risk of adverse maternal and fetal outcomes with increasing maternal glycaemia—even below the diagnostic threshold for GDM—suggesting the that criteria for intervention needed to be adjusted. The IADPSG therefore recommends that all women undergo a fasting plasma glucose (FPG) test at their first prenatal visit (where a reading ≥92 mg/dL is indicative of GDM), and that women with FPG <92 mg/dL undergo a 2-h 75 g oral glucose tolerance test (OGTT) between 24 and 28 weeks’ gestation. These glycemic cut-offs are lower than other guidelines, and only one abnormal glucose reading is required for diagnosis, which has resulted in a drastic increase in the number of cases of GDM and associated healthcare costs [12]. For this reason, there has been much discussion amongst experts as to whether the IADPSG criteria should be modified to only screen at-risk women (i.e., women of advanced maternal age, those who are overweight/obese, who are in high-risk ethnic groups, or with a family history of diabetes). However, some studies suggest that such efforts would miss a substantial number of GDM cases without significantly reducing the cost [13,14,15]. Therefore, the IADPSG criteria are the most widely recommended guideline today, although alternate criteria remain in some centers and countries (Table 1).

The inconsistencies in screening and diagnosis of GDM make worldwide estimates difficult. Using the IADPSG’s criteria, the International Diabetes Federation (IDF) estimated that 18 million live births worldwide (14%) were affected by gestational diabetes in 2017 [2]. South-East Asia had the highest prevalence of GDM at 24.2%, while the lowest prevalence was seen in Africa at 10.5%. Almost 90% of cases of hyperglycemia in pregnancy occurred in low- and middle-income countries, where access to maternal healthcare is limited. Even within-countries, GDM prevalence varies depending on race/ethnicity and socioeconomic status. Aboriginal Australians, Middle Easterners, and Pacific Islanders are the most at-risk groups for GDM [16]. Within the United States, Native Americans, Hispanics, Asians, and African-American women are at a higher risk of GDM than Caucasian women [17]. There is also some evidence that GDM prevalence varies by season, with more diagnoses of GDM in summer than winter [18].

### 1.3. Forms of Gestational Diabetes

Outside of pregnancy, three distinct forms of diabetes mellitus are described: autoimmune diabetes (type 1), diabetes occurring on a background of insulin resistance (type 2), and diabetes as a result of other causes, including genetic mutation, diseases of the exocrine pancreas (e.g., pancreatitis), and drug- or chemical-induced diabetes (such as after organ transplantation or in the treatment of human immunodeficiency virus infection and acquired immune deficiency syndrome (HIV/AIDS)) [1,19]. While there is evidence that GDM can occur in all three settings [20,21], the vast majority (~80%) of GDM cases present as β-cell dysfunction on a background of chronic insulin resistance, to which the normal insulin resistance of pregnancy is partially additive [22]. Thus, affected women tend to have an even greater degree of insulin resistance than healthy pregnant women, and therefore have further reductions in glucose utilization and increased glucose production and FFA concentrations [23]. It is thought that β-cells deteriorate due to excessive insulin production in response to excess energy consumption and insulin resistance, exhausting the cells over time. The fact that this pathology closely resembles that of T2DM has spurred much debate about whether the two diseases should be considered to be etiologically indistinct [24,25]. As this form of GDM is by far the most common, it will be the focus of this review.

### 1.4. Risk Factors for Gestational Diabetes

Epidemiological studies of risk factors for GDM are limited and are typically afflicted by confounding factors [26,27]. In addition, inconsistencies in diagnostic criteria for GDM and measurements of risk factors make it difficult to compare findings across studies. Despite these concerns, several risk factors for GDM emerge consistently. These include overweight/obesity [28], excessive gestational weight gain [29], westernized diet [30], ethnicity [31], genetic polymorphisms [32], advanced maternal age [33], intrauterine environment (low or high birthweight [34]), family and personal history of GDM [35], and other diseases of insulin resistance, such as polycystic ovarian syndrome (PCOS) [26].

Each of these risk factors are either directly or indirectly associated with impaired β-cell function and/or insulin sensitivity. For example, overweight and obesity are intrinsically linked with prolonged, excessive calorie intake, which overwhelms β-cell insulin production and insulin signaling pathways. Even independently of body mass index (BMI) and overall caloric intake, diet and nutrition are associated with GDM. Diets that are high in saturated fats, refined sugars, and red and processed meats are consistently associated with an increased risk of GDM [36,37], while diets high in fiber, micronutrients, and polyunsaturated fats are consistently associated with a reduced risk of GDM [38,39,40]. Saturated fats directly interfere with insulin signaling [41], and they can also induce inflammation and endothelial dysfunction—both pathogenic factors in GDM [42]. On the other hand, n-3 polyunsaturated fatty acids, including those derived from fish and seafood, have anti-inflammatory properties [38]. The relationship between processed meat and GDM remains strong, even after adjustment for fatty acids, cholesterol, heme iron, and protein content [43]. It has been suggested that by-products related to the processing of meat could be responsible—such as nitrates (a common preservative in processed meats), or advanced glycation end products (AGEs), which have both been implicated in β-cell toxicity [44,45]. Interestingly, even independently of meat consumption, high protein diets are associated with GDM [46,47,48]. One theory for this is the role of amino acids as substrates for hepatic glucose production [49], and in hepatic lipotoxicity [50]. The inverse association between dietary fiber and GDM may be the result of reduced appetite or slowed glucose absorption, reducing demand on β-cells and insulin signaling mediators [39].

Low and high birthweight are likely risk factors for GDM because of their association with insulin resistance. Low birthweight is often the result of undernutrition in the womb, either as a result of maternal undernutrition or placental insufficiency. It is believed that the fetus compensates for undernutrition in the womb by epigenetically altering the expression of genes that are involved in fat storage, energy utilisation, and appetite regulation. Further, animal studies suggest that undernutrition in utero is associated with reduced β-cell number [51]. These alterations persist after birth—a phenomenon referred to as “developmental programming” [52]. While potentially beneficial in times of famine, a mismatch between nutritional status in the womb and nutritional status once born can contribute to the development of obesity and metabolic disease [53,54]. On the opposite end of the spectrum, overnutrition in the womb—such as can occur in GDM—can result in fetal overgrowth. These individuals are more likely to have experienced hyperglycemia and β-cell fatigue even before birth, predisposing them to hyperglycemia during times of later metabolic stress, such as during pregnancy [55].

### 1.5. Consequences of Gestational Diabetes

The importance of aiming to understand and effectively treat or prevent GDM is illustrated by the wide-ranging consequences of GDM for both the mother and the fetus.

*Mother*—GDM increases the risk of a number of short-term and long-term maternal health issues. In addition to the stress of normal pregnancy, GDM is associated with antenatal depression [56]. There is also an increased risk of additional pregnancy complications, including preterm birth and preeclampsia, and, in many cases, surgical delivery of the baby is required [57]. Approximately 60% of women with a past history of GDM develop T2DM later in life [58]. Each additional pregnancy also confers a threefold increase in the risk of T2DM in women with a history of GDM. Further, women with a previous case of GDM have a yearly risk of conversion to T2DM of ~2 to 3% [58]. Emerging evidence also suggests that the vasculature of women with a prior case of GDM is permanently altered, predisposing them to cardiovascular disease (CVD). A recent study reported a 63% increased risk of CVD amongst women with a history of GDM, which was partly, but not fully, explained by BMI [59]. This is of major concern, as CVD is the number one cause of death in the world [60].

*Child*—GDM also poses short- and long-term consequences for the infant. The aforementioned increase in placental transport of glucose, amino acids, and fatty acids stimulate the fetus’s endogenous production of insulin and insulin-like growth factor 1 (IGF-1). Together, these can cause fetal overgrowth, often resulting in macrosomia at birth [61]. As previously mentioned, excess fetal insulin production can stress the developing pancreatic β-cells, contributing to β-cell dysfunction and insulin resistance, even prenatally [62]. Macrosomia is also a risk factor for shoulder dystocia—a form of obstructed labor. Thus, babies of GDM pregnancies are usually delivered by caesarean section [63,64]. Once delivered, these babies are at increased risk of hypoglycemia, which is likely due to formed dependence on maternal hyperglycemia (fetal hyperinsulinemia), which can contribute to brain injury if not properly managed [65]. There is also evidence that GDM increases the risk of stillbirth [66]. In the long term, babies that are born of GDM pregnancies are at increased risk of obesity, T2DM, CVD, and associated metabolic diseases. Children born to mothers with GDM have almost double the risk of developing childhood obesity when compared with nondiabetic mothers, even after adjusting for confounders such as maternal BMI [67,68], and impaired glucose tolerance can be detected as young as five years old [69]. Females are therefore more likely to experience GDM in their own pregnancies, contributing to a vicious intergenerational cycle of GDM [70].

## 2. Pathophysiology of Gestational Diabetes

The remainder of this review will discuss molecular processes underlying the pathophysiology of GDM. GDM is usually the result of β-cell dysfunction on a background of chronic insulin resistance during pregnancy and thus both β-cell impairment and tissue insulin resistance represent critical components of the pathophysiology of GDM. In most cases, these impairments exist prior to pregnancy and can be progressive—representing an increased risk of T2DM post-pregnancy [71]. A number of additional organs and systems contribute to, or are affected by, GDM. These include the brain, adipose tissue, liver, muscle, and placenta.

### 2.1. β-Cell Dysfunction

The primary function of β-cells is to store and secrete insulin in response to glucose load. When β-cells lose the ability to adequately sense blood glucose concentration, or to release sufficient insulin in response, this is classified as β-cell dysfunction. β-cell dysfunction is thought to be the result of prolonged, excessive insulin production in response to chronic fuel excess [72]. However, the exact mechanisms underlying β-cell dysfunction can be varied and complex [73,74]. Defects can occur at any stage of the process: pro-insulin synthesis, post-translational modifications, granule storage, sensing of blood glucose concentrations, or the complex machinery underlying exocytosis of granules. Indeed, the majority of susceptibility genes that are associated with GDM are related to β-cell function, including potassium voltage-gated channel KQT-like 1 (*Kcnq1*) and glucokinase (*Gck*). Minor deficiencies in the β-cell machinery may only be exposed in times of metabolic stress, such as pregnancy [75].

β-cell dysfunction is exacerbated by insulin resistance. Reduced insulin-stimulated glucose uptake further contributes to hyperglycemia, overburdening the β-cells, which have to produce additional insulin in response. The direct contribution of glucose to β-cell failure is described as glucotoxicity [76]. Thus, once β-cell dysfunction begins, a vicious cycle of hyperglycemia, insulin resistance, and further β-cell dysfunction is set in motion.

Animal studies suggest that β-cell number is also an important determinant of glucose homeostasis. For example, Zucker fatty (ZF) rats that were subjected to 60% pancreatectomy mostly recover β-cell mass by one week post-surgery, but still develop hyperglycemia. In these cases, the short-term but dramatic reduction in β-cell mass overburdens the remaining β-cells, resulting in severely reduced glucose-stimulated insulin secretion and the depletion of internal insulin granule stores [77]. Sprague Dawley rats, which are usually very resistant to the development of diabetes, experience substantial loss of β-cell mass (50% reduction) by 15-weeks old when growth-restricted in utero via bilateral uterine artery ligation [78]. This loss of β-cell mass has been linked to epigenetic downregulation of pancreatic homeobox transcription factor (*Pdx1*), which is essential for normal β-cell differentiation in the embryo [79]. Prolactin is also essential for adequate β-cell proliferation, as demonstrated in mouse knockouts of the prolactin receptor (PrlR^−/−^) [80]. In addition, glucotoxicity is also thought to result in β-cell apoptosis over time [76]. Pancreatic samples from T2DM patients can show a reduction of β-cell mass by 40–60% [81], but less than 24% loss after five years of disease has also been reported [82]. Reduced β-cell hyperplasia may also play a role in GDM, based on animal studies and limited post-mortem human studies [83]. Therefore, reduced β-cell mass, reduced β-cell number, β-cell dysfunction, or a mix of all three contribute to GDM, depending on the individual.

### 2.2. Chronic Insulin Resistance

**Figure 1:**
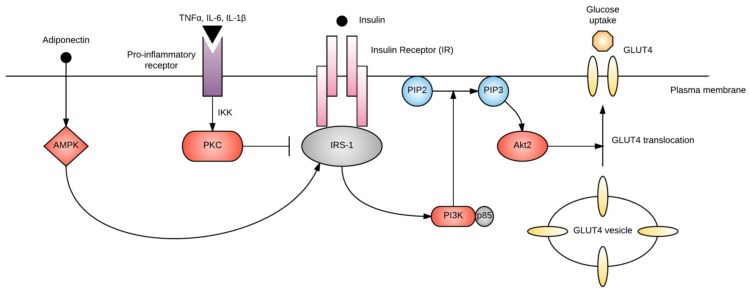
Simplified diagram of insulin signaling. Binding of insulin to the insulin receptor (IR) activates IRS-1. Adiponectin promotes IRS-1 activation through AMP-activated protein kinase (AMPK), while pro-inflammatory cytokines activate protein kinase C (PKC) via IκB kinase (IKK), which inhibits IRS-1. IRS-1 activates phosphatidylinositol-3-kinase (PI3K), which phosphorylates phosphatidylinositol-4, 5-bisphosphate (PIP2) to phosphatidylinositol-3, 4, 5-phosphate (PIP3). PIP3 activates Akt2, which promotes GLUT4 translocation and glucose uptake into the cell.

Insulin resistance occurs when cells no longer adequately respond to insulin. At the molecular level, insulin resistance is usually a failure of insulin signaling, resulting in inadequate plasma membrane translocation of glucose transporter 4 (GLUT4)—the primary transporter that is responsible for bringing glucose into the cell to use as energy (Figure 1). The rate of insulin-stimulated glucose uptake is reduced by 54% in GDM when compared with normal pregnancy [84]. While insulin receptor abundance is usually unaffected, reduced tyrosine or increased serine/threonine phosphorylation of the insulin receptor dampens insulin signaling [85]. In addition, altered expression and/or phosphorylation of downstream regulators of insulin signaling, including insulin receptor substrate (IRS)-1, phosphatidylinositol 3-kinase (PI3K), and GLUT4, has been described in GDM [84]. Many of these molecular changes persist beyond pregnancy [86].

Several of the previously discussed risk factors for GDM are thought to exert their effects by interfering with insulin signaling. For example, saturated fatty acids increase intracellular concentrations of diacylglycerol within myocytes, activating protein kinase C (PKC) and inhibiting tyrosine kinase, IRS-1 and PI3K [41]. Pro-inflammatory cytokines and adiponectin also modify this process, as discussed below.

**Figure 2:**
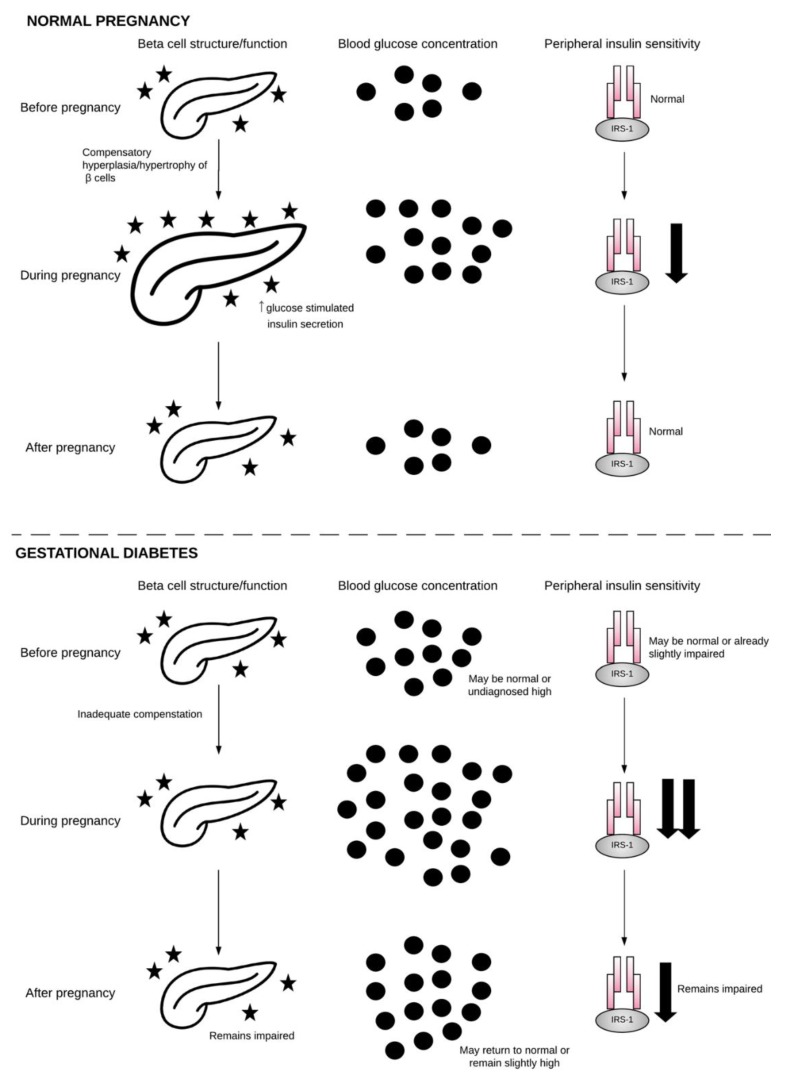
β-cell, blood glucose, and insulin sensitivity during normal pregnancy and GDM. During normal pregnancy, β-cells undergo hyperplasia and hypertrophy in order to meet the metabolic demands of pregnancy. Blood glucose rises as insulin sensitivity falls. Following pregnancy, β-cells, blood glucose, and insulin sensitivity return to normal. During gestational diabetes, β-cells fail to compensate for the demands of pregnancy, and, when combined with reduced insulin sensitivity, this results in hyperglycemia. Following pregnancy, β-cells, blood glucose, and insulin sensitivity may return to normal or may remain impaired on a pathway toward GDM in future pregnancy or T2DM. Pancreas image obtained from The Noun Project under the terms and conditions of the Creative Commons Attribution (CC BY) license (http://creativecommons.org/licenses/by/4.0/), by artist Arif Fajar Vulianto.

A diagram of the relationship between β-cell dysfunction, insulin resistance, and GDM is provided in Figure 2.

### 2.3. Neurohormonal Networks

Neurohormonal dysfunction has been implicated in the pathogenesis of diseases of insulin resistance, such as that present in GDM. This network regulates appetite, active energy expenditure, and basal metabolic rate, and it is made up of a complex network of central (e.g., cortical centers that control cognitive, visual, and “reward” cues) and peripheral (e.g., satiety and hunger hormones) signals [87,88]. These contribute to GDM by influencing adiposity and glucose utilization. This network is highly regulated by the circadian clock, which may explain why pathological sleep disorders or those individuals undertaking shift work are correlated with GDM rates [89,90]. Neural networks controlling body weight are most likely set in early life, as demonstrated in animal studies. For example, rats that are both under- and over-fed in early life experience epigenetic alteration of the regulatory set-point of hypothalamic neurons [91,92]. This adds to the previously mentioned suggestion that predisposition to GDM may be set in the womb.

Some of the most important regulators of neurohormonal metabolic control are adipokines—cell signaling proteins that are secreted primarily by adipose tissue. These include leptin and adiponectin:

#### 2.3.1. Leptin

Leptin is a satiety hormone secreted primarily by adipocytes in response to adequate fuel stores. It primarily acts on neurons within the arcuate nucleus of the hypothalamus to decrease appetite and increase energy expenditure. Specifically, leptin inhibits appetite-stimulators neuropeptide Y (NPY) and agouti-related peptide (AgRP), and it activates the anorexigenic polypeptide pro-opiomelanocortin (POMC) [93]. When leptin was first discovered, it was lauded as a potential treatment for obesity [94]. However, it was soon revealed that the majority of obese individuals do not respond to leptin, and instead demonstrate leptin resistance. While leptin treatment is effective in obesity that is caused by leptin and leptin receptor genetic polymorphisms, these are rare (<5% of obese individuals) [95]. Therefore, obesity is associated with excessive plasma leptin concentration (hyperleptinemia) as a result of leptin resistance, and plasma leptin concentrations are generally proportional to the degree of adiposity [96]. Leptin resistance can occur either as a defect in blood-brain barrier leptin transport, or through intracellular mechanisms that are similar to insulin resistance [97]. Like insulin resistance, a degree of leptin resistance occurs in normal pregnancy, presumably to bolster fat stores beyond what would usually be required in the non-pregnant state. Leptin resistance is further increased in GDM, resulting in hyperleptinemia [98]. However, pre-pregnancy BMI is a stronger predictor of circulating leptin than GDM *per se* [99].

The placenta also secretes leptin during human pregnancy. In fact, the placenta is responsible for the majority of plasma leptin during pregnancy [100]. Placental leptin production is increased in GDM, probably as a result of placental insulin resistance, and this further contributes to hyperleptinemia. This is also thought to facilitate amino acid transport across the placenta, contributing to fetal macrosomia [101].

#### 2.3.2. Adiponectin

Similar to leptin, adiponectin is a hormone that is primarily secreted by adipocytes. However, plasma adiponectin concentrations are inversely proportional to adipose tissue mass, with low concentrations in obese individuals. GDM is similarly associated with decreased adiponectin [102]. In contrast to leptin, there is a stronger association of adiponectin with insulin resistance than with adiposity [103]. This suggests that adiponectin plays an important role in the pathogenesis of GDM, independent of obesity. Adiponectin enhances insulin signaling and fatty acid oxidation, and it inhibits gluconeogenesis [104]. It does so by activating AMP-activated protein kinase (AMPK) within insulin-sensitive cells, which facilitates the action of IRS-1 (Figure 1), and by activating the transcription factor peroxisome proliferator-activated receptor alpha (PPARα) in the liver. Furthermore, adiponectin stimulates insulin secretion, by upregulating insulin gene expression and exocytosis of insulin granules from β-cells [105].

Adiponectin is also expressed at low concentration from the syncytiotrophoblast of the placenta where it is regulated by cytokines, such as tumor necrosis factor alpha (TNF-α), interleukin (IL)-6, interferon gamma (IFN-γ), and leptin [106]. The role of placental adiponectin in normal and GDM pregnancy is unclear [107]. However, emerging evidence suggests adiponectin impairs insulin signaling and amino acid transport across the placenta, limiting fetal growth. Therefore, adiponectin gene methylation in the placenta is associated with maternal glucose intolerance and fetal macrosomia [108].

### 2.4. Adipose Tissue

Originally believed to exist only as a passive depot of energy, the discovery of leptin in 1994 established adipose tissue as an essential endocrine organ. Adipose tissue both ensures that energy is partitioned safely and it actively secretes circulatory factors, including adipokines (the aforementioned leptin and adiponectin) and cytokines (such as TNF-α, IL-6, and IL-1β), which have wide-ranging metabolic effects.

#### 2.4.1. Energy Storage

The storage capability of adipose tissue is essential for metabolic health. This is exemplified through two extremes: rare disorders in which white adipose tissue is absent lead to severe metabolic syndrome, whereas some obese individuals (with excessive white adipose tissue) do not develop metabolic syndrome at all [109]. Therefore, the ability to partition excess calories into adipose tissue rather than ectopically in the liver, muscle, or pancreas, appears to serve as a protective measure. Non-diabetic obese individuals exhibit adequate adipose tissue expansion in response to fuel surfeit, and therefore maintain healthy blood glucose concentrations, sufficient β-cell compensation, and avoid chronic insulin resistance [110,111]. In this way, key organs avoid glucose and fatty acid-induced tissue damage. As previously mentioned, early pregnancy is marked by an increase in adipose tissue mass, while later pregnancy promotes the mobilization of fats from adipose tissue in order to fuel fetal growth. Both of these processes are thought to be limited in GDM [112]. GDM is associated with reduced adipocyte differentiation and increased adipocyte size (hypertrophy), accompanied by downregulated gene expression of insulin signaling regulators, fatty acid transporters, and key adipogenic transcription factors, such as PPARγ [113]. The combination of insulin resistance and reduced adipocyte differentiation hinders the tissue’s ability to safely dispose of excess energy, contributing to gluco- and lipo-toxicity in other peripheral organs. Indeed, both T2DM and GDM are associated with lipid deposition in muscle and liver [114,115].

#### 2.4.2. Adipose Tissue Inflammation

Obesity, T2DM and GDM are associated with an increased number of resident adipose tissue macrophages (ATM) that secrete pro-inflammatory cytokines, including TNF-α, IL-6, and IL-1β. The importance of a low-grade inflammatory state in the pathogenesis of insulin resistance has recently become apparent. Pro-inflammatory cytokines have been discovered to both impair insulin signaling and inhibit insulin release from β-cells. These factors induce insulin resistance either by diminishing insulin receptor (IR) tyrosine kinase activity, increasing serine phosphorylation of IRS-1, or through the STAT3-SOCS3 pathway, which degrades IRS-1 [85,116]. Circulating concentrations of pro-inflammatory cytokines are increased in GDM [107,117]. Plasma TNF-α, in particular, is strongly correlated with insulin resistance [118]. Similarly, placental gene expression of TNF-α, IL-1β and their receptors has been reported to be increased in GDM [118,119]. However, the relationship between pregnancy and inflammation is complex. For example, Lappas et al. (2010) reported that GDM placentae secrete *fewer* pro-inflammatory cytokines (3 of 16 studied: IL-1β, TNF-α and M1P1B) than healthy placentae (13 out of 16 studied) [120]. This suggests that, while chronic low-grade inflammation appears to be important in the pathogenesis of GDM, the relationship may not be straightforward.

### 2.5. Liver

GDM is associated with upregulated hepatic glucose production (gluconeogenesis). Gluconeogenesis is increased in the fasted state, and not adequately suppressed in the fed state [84]. This is not believed to be entirely the result of inaccurate glucose sensing due to insulin resistance, as the majority of glucose uptake by the liver (~70%) is not insulin dependent. Common factors between the insulin signaling pathway and the pathways controlling gluconeogenesis, such as PI3K, might contribute to these effects [121]. Increased protein intake and muscle breakdown may also stimulate the process by providing excess gluconeogenesis substrate [122]. Despite this, the liver does not seem to be a primary pathogenic driver of T2DM or GDM [123].

### 2.6. Skeletal and Cardiac Muscle

Traditionally, skeletal muscle insulin resistance was believed to play a causal role in T2DM. However, skeletal muscle insulin resistance now appears to be a consequence of hyperglycemia—a protective measure to prevent metabolic stress and steatosis [124]. Even following a short period of overfeeding, cardiac and skeletal muscle develop insulin resistance in order to divert the excess energy into adipose tissue [125]. This is an important distinction when considering potential treatments for GDM: attempts to directly reverse skeletal muscle insulin resistance, without reducing plasma glucose concentrations, could be detrimental [123].

Separate to insulin sensitivity, T2DM and GDM are associated with a reduced number and function of mitochondria within skeletal muscle cells [126]. This could be the result of genetics, early-life programming, or chronic inactivity. Therefore, decreased number and function of mitochondria is likely an additional contributor to reduced glucose utilization in GDM.

### 2.7. Gut Microbiome

There is emerging evidence that microbial organisms within the gut—the “gut microbiome”—might contribute to metabolic diseases, including GDM. The gut microbiome can be influenced by early-life events, such as preterm delivery and breastfeeding, and by events in later life, such as diet composition and antibiotic use. The gut microbiome has been consistently reported to differ between metabolically healthy and obese individuals, including during pregnancy [127]. Furthermore, a study of stool bacteria in women with a past case of GDM reported a lower proportion of the phylum *Firmicutes* and higher proportion of the family *Prevotellaceae* as compared with normoglycemic pregnancy [128]. Similar associations have been observed in obesity [129], T2DM [130], fatty liver disease [131], and elevated total plasma cholesterol [132]. *Firmicutes* metabolize dietary plant polysaccharides. This may explain some of the dietary risk factors for GDM that are discussed earlier. Both red meat and animal protein decrease levels of *Firmicutes*, while high dietary fiber increase them [133]. However, the findings by Fugmann et al. (2015) remained after adjustment for dietary habits [128]. Therefore, *Firmicutes* appear to be relevant to pathogenesis of GDM independent of diet, although the mechanisms underlying this are unknown. *Prevotellaceae* are mucin-degrading bacteria that may contribute to increased gut permeability. Gut permeability is regulated by tight junction proteins, such as zonulin (ZO-1). Increased “free” plasma/serum ZO-1 is associated with type 1 diabetes (T1DM), T2DM [134], and GDM [135]. Increased gut permeability is thought to facilitate the movement of inflammatory mediators from the gut into the circulation, promoting systemic insulin resistance [134,136].

### 2.8. Oxidative Stress

Oxidative stress describes an imbalance between pro-oxidants and antioxidants in cells. Oxidative stress can lead to cellular damage by interfering with the state of proteins, lipids and DNA, and has been implicated in the pathogenesis of many diseases, including GDM [137]. Reactive oxygen species (ROS) are described as free radical and nonradical derivatives of oxygen, and include superoxide anion (O_2_^−^), hydroxyl radical (•OH) and hydrogen peroxide (H_2_O_2_) [138]. A hyperglycemic environment is associated with oxidative stress, and GDM women have been reported to overproduce free radicals and have impaired free-radical scavenging mechanisms [139]. ROS inhibit insulin-stimulated glucose uptake by interfering with both IRS-1 and GLUT4 [140]. ROS also slow glycogen synthesis in the liver and muscle. Pro-inflammatory cytokines, such as TNF-α, may also contribute to oxidative stress by increasing the expression and the activation of ROS precursors, like NADPH oxidase 4 (NOX4) [141].

Interestingly, iron supplementation in women already replete in iron is associated with GDM [142]. Several studies suggest that this relationship is the result of increased oxidative stress. Iron is a transitional metal and it can catalyze the reaction from O_2−_ and H_2_O_2_ to the extremely reactive •OH within mitochondria [143]. On the contrary, selenium and zinc are transitional metals that are necessary for the activity of some antioxidant enzymes, which may explain their inverse association with GDM [144].

Homocysteine—a non-protein α-amino acid that is formed by the demethylation of methionine—is also thought to contribute to GDM via oxidative stress. Exposure of β-cells to even small amounts of homocysteine results in dysfunction and impaired insulin secretion [145]. A recent meta-analysis examined the relationship between serum homocysteine concentration and GDM in ten eligible studies. The authors reported significantly higher homocysteine concentrations among women with GDM as compared with those without GDM [146]. B vitamins, including folic acid, B2, B6, and B12 are essential for homocysteine homeostasis, and this may be one reason why deficiencies and imbalances of these micronutrients are associated with GDM [147].

### 2.9. Placental Transport

The placenta contributes to insulin resistance during pregnancy via its secretion of hormones and cytokines. As the barrier between the maternal and fetal environments, the placenta itself is also exposed to hyperglycemia and its consequences during GDM. This can impact transport of glucose, amino acids, and lipids across the placenta:

*Glucose—*Glucose is the primary energy source for the fetus and the placenta, and therefore must be readily available at all times. For this reason, insulin is not required for the placental transport of glucose. Instead, glucose transport occurs via GLUT1, by carrier-mediated sodium-independent diffusion [148]. However, the placenta still expresses the insulin receptor, and insulin signaling can influence placental metabolism of glucose [149]. The receptiveness of the placenta to glucose uptake means that it is particularly sensitive to maternal hyperglycemia, and this directly contributes to increased fetal growth and macrosomia.

*Protein—*Amino acid transport across the placenta is also an important determinant of fetal growth. GDM is associated with increased System A and L activity [150]. These can also be modulated by pro-inflammatory cytokines, such as TNF-α and IL-6 [151]. Altered amino acid transport may also be one mechanism by which excess protein intake contributes to GDM.

*Lipids—*Finally, while GDM has traditionally been described as a disease of hyperglycemia, the rise in obesity-associated GDM has prompted a greater focus on the role of hyperlipidemia in GDM. The majority of placental gene expression alterations in GDM occur in lipid pathways (67%), as compared with glucose pathways (9%) [152]. Preferential activation of placental lipid genes is also associated with GDM compared with T1DM [152]. These data correlate with the results of the HAPO Study, which revealed independent effects of maternal obesity and glucose on excessive fetal growth [153]. Therefore, it appears that GDM influences the placental transport of glucose, amino acids, and fatty acids, and that all three must be considered when discussing the impact of GDM on placental function and fetal growth.

**Figure 3:**
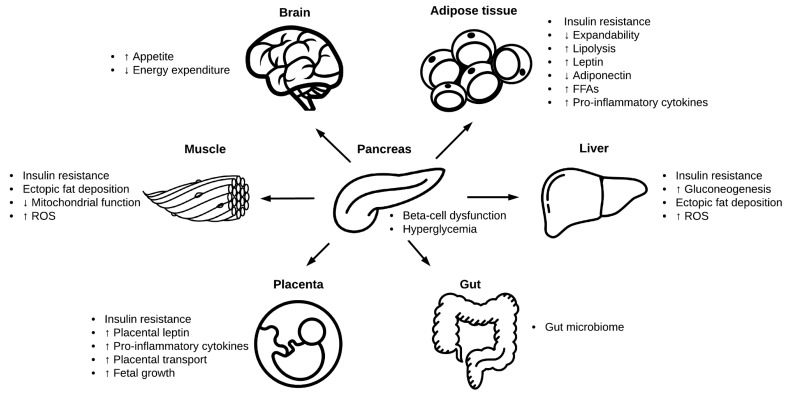
Organs involved in the pathophysiology of GDM (Images in this figure were obtained from The Noun Project under the terms and conditions of the Creative Commons Attribution (CC BY) license (http://creativecommons.org/licenses/by/4.0/). Brain and Gut by Hunotika; Liver by Lavmik; Pancreas by Arif Fajar Vulianto; Placenta by Charmeleon Design; Muscle by Misha Petrishchev).

In addition to these alterations in placental transport, GDM has been associated with other changes in the placenta. Some recent studies have reported that GDM is associated with placenta global DNA hypermethylation [154]. Similarly, studies of the placental proteome have identified differences in the expression of proteins between GDM and non-GDM placentas [155]. However, more research is required before the role of placental epigenetic and proteomic modifications in GDM is fully understood [156]. There has also been recent interest in small noncoding single-stranded segments of RNA, called microRNAs (miRNAs), expressed in placental trophoblast cells. miRNAs are involved in a number of cellular processes, including proliferation, differentiation, and apoptosis. Emerging evidence suggests that exosomes containing miRNAs are shed from the placenta during gestation and released into the maternal circulation, which can in turn influence the functioning of other cells, potentially contributing to the pathogenesis of GDM [157,158]. Interestingly, exposure to endocrine disrupting chemicals (EDCs), including bisphenol A (BPA—found in food packaging materials and consumer products) has been associated with GDM, and it has been suggested that this could be because EDCs induce exosome signaling from the placenta [159]. Interestingly, EDCs including BPA have also been associated with alterations in methylation, perhaps linking the two mechanisms [160]. A summary diagram of the pathophysiology of GDM is presented in Figure 3.

## 3. Opportunities and Considerations for Future Study

Uncovering the intricate molecular mechanisms underlying GDM is challenging, but necessary for our greater understanding of the disease and how these could assist in the design of new treatments. As β-cell dysfunction and insulin resistance are the hallmarks of GDM, the greatest emphasis should be placed on further understanding the mechanisms underlying these processes. For example, why do β-cells exhibit proper hyperplasia and hypertrophy in some pregnancies, but not others? How could we modify these processes to bolster pancreatic function and prevent hyperglycemia in at-risk individuals? As already mentioned, increasing insulin sensitivity could have unintended consequences by promoting uptake of glucose into tissue where energy should not be stored, such as the liver and skeletal muscle. Instead, investment into adipose-specific insulin sensitivity should be examined. While improving adipose capacity (and in theory increasing adipose tissue mass) might seem counterintuitive, in actuality, it should reduce hyperglycemia while ensuring that excess energy is stored safely. Of course, many of the mechanisms underlying GDM are not unique to GDM, encompassing other common disorders of insulin sensitivity, such as T2DM, prediabetes, and PCOS. Therefore, determination of pathways influencing development of these metabolic disorders may also shed light on GDM, and potentially accelerate opportunities for prevention and/or treatment. This is an important consideration, as the study of GDM (as a disorder of pregnancy) is limited for ethical reasons. Finally, the ability to study large amounts of data through computer technology is rapidly advancing the fields of genomics, epigenetics, proteomics, metagenomics (the microbiome), and metabolomics (the study of the small-molecule intermediates and products of metabolism). It is hopeful that the advancement of these large-scale techniques may assist in our understanding of the pathogenesis of GDM in the future.

## 4. Conclusions

Pregnancy is a state of high metabolic activity, in which maintaining glucose homeostasis is of upmost importance. When hyperglycemia is detected in the pregnant mother, this is referred to as GDM, although controversy remains over diagnostic criteria. It is likely that genetic, epigenetic, and environmental factors all contribute to the development of GDM, and that the mechanisms involved are complex and advance over a substantial period of time. However, in the majority of cases, pancreatic β-cells fail to compensate for a chronic fuel surfeit, leading to eventual insulin resistance, hyperglycemia, and an increased supply of glucose to the growing fetus. There is also evidence that adipose expandability, low-grade chronic inflammation, gluconeogenesis, oxidative stress, and placental factors contribute to the pathology of GDM. Greater understanding of these processes and their contribution to GDM is required in order to develop effective treatments and prevention strategies.

## Figures and Tables

**Table 1:** Various criteria for gestational diabetes mellitus (GDM) diagnosis using oral glucose tolerance test (OGTT).

Criteria	Pregnancies	Timing of OGTT	Steps	Glucose Load (g)	Glucose Threshold (mmol/L)			
					Fasting	1 h	2 h	3 h
O’Sullivan, 1964	All	24–28 weeks	2	100	5.0	9.2	8.1	6.9
WHO, 1999	All	24–28 weeks	1	75	7.0	—	7.8	—
American Diabetes Association (ADA), 2004	High and medium risk	14–18 weeks for high risk, 28–32 weeks for medium risk	2	100	5.3	10.0	8.6	7.8
National Institute for Health and Care Excellence (NICE), 2015	High risk	As early as possible	1	75	5.6	—	7.8	—
IADPSG, 2010	All	24–28 weeks	1	75	5.1	10.0	8.5	—
WHO, 2013								
ADA, 2016								
